# A Good Deodoriser

**Published:** 1916-03-25

**Authors:** G. Arbour Stephens

**Affiliations:** 61 Walter Road, Swansea


### THE EDITOR'S LETTER-BOX.

**Our Correspondents are reminded that brevity of style and conciseness of statements greatly facilitate early insertion. We cannot in any way be responsible for the opinions expressed by correspondents, who must give their name and address as a guarantee of good faith, but not necessarily for publication.**

### A Good Deodoriser.

*To the Editor of* The Hospital.

Sir,—It may be of interest to those who have to do with patients suffering from diseases associated with foul discharges or offensive stools to know that the use of a petrol spray immediately removes the objectionable smell.

A very small quantity of this universally used commodity is sufficient to produce the required effect. In a very bad case of colotomy where the smell was so bad as to make it difficult to approach the bed a few blows of the spray enabled one to do it comfortably and to remain without any inconvenience. Its use in cleaning wounds and burns has been pointed out in the *Dublin Journal of Medical Science, Lancet,* and *British Medical Journal,* but as a deodoriser for the benefit of nurses this is the first time it has been mentioned.

It seems to act on the minute but solid particles which are responsible for the smell, and by affecting their surface tension cause them, as it were, to fall down. Unlike other deodorisers, it does not substitute one strong smell for another, whereby the connection in after-times has a marked mental associational effect of an unpleasant nature, but gives one the pleasing idea of cleanliness.—Yours faithfully,

G. Arbour Stephens, M.D., B.S., B.Sc. (Lond.).

61 Walter Road, Swansea, March 20, 1916.

